# Response by Sex in Patient-Centered Outcomes With Baroreflex Activation Therapy in Systolic Heart Failure

**DOI:** 10.1016/j.jchf.2021.01.012

**Published:** 2021-05-12

**Authors:** JoAnn Lindenfeld, Richa Gupta, Luanda Grazette, Jean Marie Ruddy, Lana Tsao, Elizabeth Galle, Tyson Rogers, Samuel Sears, Faiez Zannad

**Affiliations:** a Division of Cardiology, Vanderbilt University Medical Center, Nashville, Tennessee, USA; b Keck School of Medicine, University of Southern California, Los Angeles, California, USA; c Medical University of South Carolina, Charleston, South Carolina, USA; d Saint Elizabeth’s Medical Center, Boston, Massachusetts, USA; e CVRx, Minneapolis, Minnesota, USA; f NAMSA, Minneapolis, Minnesota, USA; g East Carolina State University, Greenville, North Carolina, USA; h Inserm Centre d’Investigation, CHU de Nancy, Institute Lorrain du Coeur et des Vaisseaux, Université de Lorraine, Nancy, France

**Keywords:** baroreflex, congestive, heart failure

## Abstract

**OBJECTIVES:**

The aim of this study was to assess sex differences in the efficacy and safety of baroreflex activation therapy (BAT) in the BeAT-HF (Baroreflex Activation Therapy for Heart Failure) trial.

**BACKGROUND:**

Patients were randomized 1:1 to receive guideline-directed medical therapy (GDMT) alone (control group) or BAT plus GDMT.

**METHODS:**

Pre-specified subgroup analyses including change from baseline to 6 months in 6-min walk distance (6MWD), quality of life (QoL) assessed using the Minnesota Living With Heart Failure Questionnaire (MLWHQ), New York Heart Association (NYHA) functional class, and N-terminal pro–B-type natriuretic peptide (NT-proBNP) were conducted in men versus women.

**RESULTS:**

Fifty-three women and 211 men were evaluated. Women had similar baseline NT-proBNP levels, 6MWDs, and percentage of subjects with NYHA functional class III symptoms but poorer MLWHQ scores (mean 62 ± 22 vs. 50 ± 24; p = 0.01) compared with men. Women experienced significant improvement from baseline to 6 months with BAT plus GDMT relative to GDMT alone in MLWHQ score (—34 ± 27 vs. —9 ± 23, respectively; p < 0.01), 6MWD (44 ± 45 m vs. —32 118 m; p < 0.01), and improvement in NYHA functional class (70% vs. 27%; p < 0.01), similar to the responses seen in men, with no significant difference in safety. Women receiving BAT plus GDMT had a significant decrease in NT-proBNP (—43% vs. 7% with GDMT alone; difference —48%; p < 0.01), while in men this decrease was —15% versus 2%, respectively (difference —17%; p = 0.08), with an interaction p value of 0.05.

**CONCLUSIONS:**

Women in BeAT-HF had poorer baseline QoL than men but demonstrated similar improvements with BAT in 6MWD, QoL, and NYHA functional class. Women had a significant improvement in NT-proBNP, whereas men did not. (Baroreflex Activation Therapy for Heart Failure [BeAT-HF]; NCT02627196) (J Am Coll Cardiol HF 2021;9:430–8)

Heart failure with reduced ejection fraction (HFrEF) affects more than 3 million people in the United States and 13 million people worldwide (1), with women accounting for 36% of incident HFrEF and 40% of patients hospitalized for HFrEF (2,3). Compared with men, women are more symptomatic, have more evidence of volume overload, have lower health-related quality of life (QoL), and greater impairment in activities of daily living (4–7). Despite this higher symptom burden, women are systematically underenrolled in trials of pharmacological and device therapies for heart failure (HF).

In fact, only 20% to 25% of subjects in randomized HF clinical trials are women (8,9). Disparities in sex extend to access to therapies; women are undertreated with devices and less likely to receive influenza vaccination, be enrolled in disease management programs, or be prescribed exercise regimens (9).

In HF trials that have published sex-specific results, both sexes respond similarly to standard HF treatments such as angiotensin-converting enzyme inhibitors, angiotensin receptor blockers, sacubitril/valsartan, beta-blockers, mineralocorticoid receptor antagonists, and sodium glucose transport protein–2 inhibitors (9–13). However, women with HF consistently have worse QoL compared with their male counterparts (14). Thus, it is particularly important to understand the benefit of HF therapies on QoL in women.

Baroreflex activation therapy (BAT) is a form of autonomic modulation that involves stimulation of the carotid baroreceptors through an electrode attached to the bifurcation of the carotid artery. The BAROSTIM NEO system (CVRx, Minneapolis, Minnesota) consists of a pulse generator and carotid sinus lead implanted surgically to deliver BAT. During implantation, the carotid sinus is exposed and stimulated to assess the maximum heart rate and blood pressure response, and the lead is placed over the site of maximum response (15–17). The intensity of BAT is progressively up-titrated over the first 3 months of therapy, primarily by increasing electric pulse amplitude (15–17). This stimulation invokes a reduction of sympathetic activity and an increase in parasympathetic activity, resulting in increased arterial and venous compliance and reduced peripheral resistance. The therapy has been shown to be safe and effective in patients with resistant hypertension and HFrEF (15–17) and addresses the continuing unmet need for the ≥70% of patients with HFrEF ineligible for cardiac resynchronization therapy (CRT) (18). In the BeAT-HF (Baroreflex Activation Therapy for Heart Failure) trial, 245 patients were randomized to BAT versus usual therapy and followed for 6 months (19). BAT was safe and significantly improved 6-min walk distance (6MWD) by 60 m (p < 0.01), QoL by 14 points (p < 0.01), and N-terminal pro–B-type natriuretic peptide (NT-proBNP) by 25% relative reduction (p < 0.01) at 6 months (19). The purpose of this post hoc study was to assess both the benefits and safety of BAT in BeAT-HF in women compared with men.

## METHODS

### TRIAL AND PARTICIPANTS.

BeAT-HF was a prospective, multicenter trial that randomized patients with HFrEF 1:1 to receive either guideline-directed medical therapy (GDMT) for HF or GDMT with BAT with the BAROSTIM NEO system (19). The trial was designed through the Breakthrough Devices Program of the U.S. Food and Drug Administration Center for Devices and Radiological Health, which provides a pathway that enables market access for promising technologies intended to treat chronically ill patients with severe unmet needs (20).

An overview of the trial design and results have been previously published (19). In summary, patients were eligible for the trial if they had moderate to severe chronic HF, defined as New York Heart Association (NYHA) functional class III or II (with a recent history of NYHA functional class III), left ventricular ejection fraction ≤35%, and current treatment with stable GDMT for HF. Initially, an additional eligibility criterion was the presence of NT-proBNP >1,600 pg/ml in patients who had not had HF hospitalizations within the previous 12 months. This eligibility criterion was subsequently revised to exclude all patients with NT-proBNP >1,600 pg/ml. This was the final definition of the intended-use group as approved by the Food and Drug Administration that is reported in this study. Exclusion criteria included a Class 1 indication for a CRT defibrillator according to American Heart Association, American College of Cardiology, and Heart Rhythm Society guidelines for the management of HF (21). The protocol conformed to the Declaration of Helsinki and was approved by the appropriate Institutional Review Boards and ethics committees in the United States and the United Kingdom. All patients provided written informed consent at enrollment.

### OUTCOMES AND MEASURES OF CLINICAL RESPONSE.

The primary safety objective was freedom from system- and procedure-related major adverse neurologic and cardiovascular events (MANCE) in all patients implanted. The effectiveness endpoints were a change from baseline to 6 months in 6MWD, QoL as assessed by the Minnesota Living With Heart Failure Questionnaire (MLWHQ), and NT-proBNP. NYHA functional class and the EuroQol 5-Dimension Long (EQ-5D) tool were also analyzed at these time points. From the MLWHQ, both a physical and an emotional dimension were analyzed using subsets of the 21 questions. From the EQ-5D, the 5 individual dimensions and the overall health status (from 0 to 100, where 100 is best) were analyzed. Clinically relevant measures of response such as improvement in 6MWD by >10%, improvement in 6MWD by >20%, improvement in QoL by >5 points, improvement in QoL by >10 points, improvement in NYHA functional class by ≥1 class, and improvement to NYHA functional class I were also assessed. Super-responders were defined by 6-month improvement in 6MHW of >20%, improvement in MLWHQ score by >10 points, or improvement to NYHA functional class I. Sex was identified by the site reporting sex as either or “women.” “men”

### STATISTICAL METHODS.

Analyses by sex for the effectiveness endpoint were specified in the statistical analysis plan for formal evaluation, separate from other subgroup analyses, with a planned alpha level of 0.15 for the interaction of sex and treatment. Effectiveness endpoints were examined using an analysis of covariance linear regression model that included treatment group, sex, the interaction of sex and randomized arm, and the baseline value as a continuous covariate, to compare with the mean improvement from baseline to 6 months in the BAT plus GDMT group versus the GDMT alone group by sex. The mean change in NT-proBNP was analyzed on the log_10_ scale, and using an inverse transformation, change from baseline was interpreted as a comparison of the percentage change in NT-proBNP. Responder outcomes were analyzed using 2-sample *z* tests for proportions. For additional outcomes assessed in both arms, comparisons between men and women were performed using linear or logistic regression models, which included main and interaction terms for sex and randomized arm. Interaction p values were calculated using the Wald method. MANCE were assessed in the BAT group only and were compared between men and women using logistic regression.

## RESULTS

In the BeAT-HF trial, 264 patients were randomized (134 GDMT, 130 BAT plus GDMT), of whom 245 completed the 6-month visit. Of the 264 randomized subjects, 53 (20%) were women and 211 (80%) were men.

### BASELINE CHARACTERISTICS.

Women had lower body mass index, had shorter QRS durations, and were less likely to have coronary artery disease, particularly a history of coronary artery bypass surgery (Table 1). In BeAT-HF, baseline HF treatment was similar between men and women, as was the percentage with implantable cardioverter-defibrillators (ICDs). Importantly, baseline NT-proBNP was similar between sexes, yet women had worse QoL as measured using both the MLWHQ and EQ-5D. Women scored more poorly on both the physical emotional domains of the MLWHFQ and at baseline felt worse about their overall health and had greater anxiety and depression compared with men (Table 2).

### EFFECTIVENESS ENDPOINTS.

The Central Illustration shows results of the change from baseline to 6 months in the effectiveness endpoints (6MWD, MLWHFQ score, and NT-proBNP) as well as NYHA functional class by sex, including the interaction p value. For all 4 endpoints depicted, women in the BAT plus GDMT group had greater improvement from baseline to 6 months compared with those in the GDMT alone group. However, between-sex differences in 6MWD, MLWHFQ score, NYHA functional class, and NT-proBNP were not statistically significant from baseline to 6 months.

### EFFECTIVENESS ENDPOINTS: QoL DOMAINS.

BAT plus GDMT was associated with significant improvement in several QoL domains in both women and men (Table 3). In both the physical and emotional domains of the MLWHFQ, women improved significantly more from baseline to 6 months with BAT plus GDMT compared with GDMT alone. In all subcategories of the MLWHFQ and EQ-5D, there were no significant differences between sexes, but there were trends suggestive of greater benefit in women (except mobility, for which the trend favored men). Women reported more depression than men at baseline (p < 0.01) (Table 2), and 70% of women with BAT plus GDMT reported improvement in anxiety or depression at 6 months compared with 38% of men with BAT plus GDMT (interaction p = 0.12).

### CLINICALLY RELEVANT MEASURES OF RESPONSE.

**FIGURE 1:**
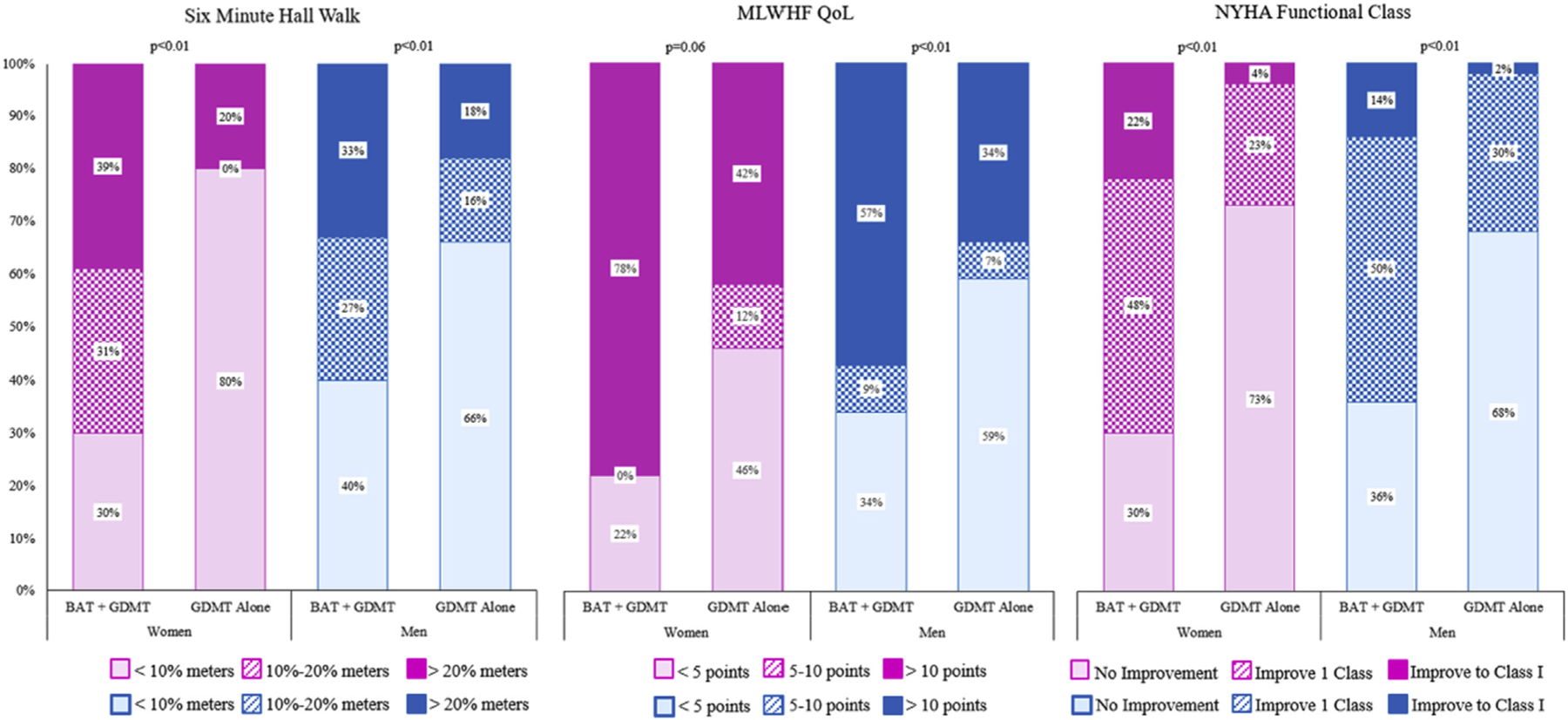
6-Month Responder Rates by Sex Both women and men showed significantly higher response rates across all symptomatic endpoints with baroreflex activation therapy (BAT) plus guideline-directed medical therapy (GDMT) compared with GDMT alone except for improvement in quality of life (QoL) >5 points, improvement in 6-min walk distance >20%, and improvement to New York Heart Association (NYHA) functional class I among women. There were no significant interaction p values by sex. See Supplemental Table S1 for additional detailed results. MLWHF = Minnesota Living With Heart Failure Questionnaire.

Clinically relevant measures of response are depicted in Figure 1, with a more detailed breakdown of these endpoints summarized in Supplemental Table S1. Both women and men showed significantly higher response rates across all symptomatic endpoints with BAT plus GDMT compared with GDMT alone, except for response rates for women for QoL improvement >5 points, 6MWD improvement >20%, and improvement to NYHA functional class I. There were no significant differences in these measures by sex.

### SAFETY ENDPOINTS.

BAT was safe in both men and women. The MANCE-free rate was 97% in men (n = 101 implanted) and 96% in women (n = 24 implanted) (p = 0.57) (Supplemental Table S2). These events specifically were decompensated HF (n = 1 man), stroke (n = 1 man), and infection in the neck requiring explantation (n = 2, 1 man and 1 woman) (Supplemental Table S3). In each case, the subject recovered with no residual effects. Serious related adverse events within 6 months of implantation were similar in women and men who received BAT, with 2 events in 1 woman (4%) and 7 events in 6 men (6%) (p = 0.75) (Supplemental Table S4). Of all patients randomized in the trial, the rate of serious unrelated adverse events in the first 6 months of follow-up was 19% in women compared with 27% in men (p = 0.21).

### CONCORDANCE WITH PHASE 2 STUDY.

Safety and effectiveness observed in the BeAT-HF study are consistent with the phase 2 study in patients with HFrEF who did not receive CRT at baseline, despite the small number of women randomized (n = 14 completed the 6-month visit, 7 underwent implantation). When combining women across the phase 2 study and BeAT-HF (n = 63 women), the pooled improvement observed in 6MWD (85 m; p < 0.001), QoL (24 points; p < 0.001), and NYHA functional class remained significant (36%; p < 0.01), and NT-proBNP reached statistical significance (54% relative reduction; p < 0.001). Note that although there were statistically significant improvements in QoL and NT-proBNP in both men and women, the interaction terms for QoL and NT-proBNP were statistically significant (p = 0.03 and p = 0.01, respectively), suggesting that women had greater improvements in QoL and NT-proBNP than observed in men. From a safety perspective, the MANCE-free rates combined in the 2 studies were similar (97% for women and 96% for men; p = 0.83). The pooled serious related adverse event rates differed more, with a 3% event rate for women and a 10% event rate for men (p = 0.11), and for serious unrelated events, the difference observed remained consistent, with a 20% event rate for women and a 30% event rate for men (p = 0.07).

## DISCUSSION

This comparison of results by sex in the BeAT-HF prospective randomized controlled trial highlights 4 important findings. First, women with HFrEF have worse QoL at baseline compared with men as measured by the physical and emotional domains of the MLWHQ, as well as greater anxiety and depression scores as assessed using the EQ-5D. Second, women had significant improvements in these QoL dimensions, 6MWD, and NYHA functional class, responding favorably to BAT, similar to men. Third, in this small sample, BAT decreased NT-proBNP levels in women relative to GDMT alone from baseline to 6 months and at least as much as in men. Finally, BAT is safe in women with HFrEF, and safety was not significantly different from that among men.

### THE BURDEN OF SYMPTOMS IN WOMEN.

Despite lower death and hospitalization rates in women with HF compared with men, women continue to experience higher symptom burden and poorer QoL. For example, an analysis of 2 large contemporary HFrEF trials revealed that women remained less likely to die than men even after adjusting for NT-proBNP and other prognostic variables (9). In this same analysis, women with HFrEF had lower Kansas City Cardio-myopathy Questionnaire clinical summary scores at baseline than men (interquartile range: 53.4 to 86.5 vs. 65.1 to 92.7; p < 0.001) and more symptoms, including dyspnea at rest and exertion, orthopnea, and edema (9). In the present study, baseline QoL by both the physical and emotional domains of the MLWHQ and the EQ-5D, as well as anxiety and depression scores, were significantly worse in women with HFrEF compared with men, which is consistent with the few HFrEF clinical trials that have studied this. The AdaptResponse trial is a contemporary CRT trial that notably recruited the highest percentage (43%) of female patients with HFrEF of any CRT trial thus far (1,569 women and 2,051 men) (22). Here, the investigators found that compared with men, women more often had advanced HF symptoms (55.6% of women were classified in NYHA functional class III or IV compared with 48.7% of men; p < 0.001), were more often depressed (18.5% vs. 9.7%; p < 0.001), and scored significantly lower on measures of QoL (61.9 ± 20.1 vs. 64.2 ± 9.3 [p < 0.001] for the EQ-5D and 57.9 ± 23.7 vs. 65.9 ± 3.3 [p < 0.001] for the Kansas City Cardiomyopathy Questionnaire) despite having fewer comorbidities and less ischemic cardiomyopathy. The disconnect between worse symptoms and QoL despite lower mortality in women compared with men remains poorly understood, in part because these baseline differences remain understudied. The present study adds to the growing body of research on baseline QoL in women with HFrEF and prompts considerations about possible differences between men and women on pathophysiological substrates (e.g., macrovascular vs. microvascular disease, hypertension), social stressors (e.g., absence of a caregiver), economic stressors, and differences in access and use of technologies such as devices (14).

### COMPARISON WITH CRT RESPONSE IN WOMEN.

In the present analysis, despite having lower baseline QoL, women improved in all major endpoints reflective of QoL, functional status, and exercise capacity, including QoL as measured by the MLWHQ and EQ-5D, 6MWD, and NYHA functional class. This also parallels findings in CRT studies, in which women experience improvements in QoL and exercise capacity measures as least as much as men. In one meta-analysis of sex differences in CRT outcomes, the investigators identified and pooled data from 8 observational studies that evaluated NYHA functional class, 4 that assessed 6MWD and 4 that reported change in QoL (23). Women were found to significantly improve in all 3 measures at both short- and long-term follow-up. This degree of improvement in QoL and 6MWD was not significantly different from that of men in either the short or long term. Although NYHA functional class improvement in the short term was not different from that of men, women had significantly more improvement in exercise capacity compared with men in the long-term follow-up period. Our analysis takes these findings even further, showing that women who received BAT plus GDMT experienced improvement in more granular parameters of QoL that have not previously been well described. These include improvements in anxiety and depression, usual activities, and self-care from baseline to 6 months compared with control. Perhaps the confirmation of symptom burden and the introduction of a novel technology provided a sense of hope and motivation to re-engage in the daily activities of life and created enhanced benefits.

The present analysis also found that women had at least as much decrease in NT-proBNP levels from baseline to 6 months compared with men. NT-proBNP is highly predictive of outcome. In the PARADIGM-HF (Prospective Comparison of ARNI with ACEI to Determine Impact on Global Mortality and Morbidity in Heart Failure) trial, for example, morbidity and mortality were reduced when the level fell by just 10% (10), and in the GUIDE-IT (Guiding Evidence Based Therapy Using Biomarker Intensified Treatment in Heart Failure) trial, reduction from >1,000 to <1,000 pg/ml was associated with increased ejection fraction and reduced left ventricular end-diastolic volume (24). Finally, in an analysis of patients enrolled in the MADIT-CRT (Multicenter Automatic Defibrillator Implantation Trial With Cardiac Resynchronization Therapy), those with CRT defibrillators in whom 1-year brain natriuretic peptide levels were reduced or remained low experienced significantly lower risk for subsequent HF or death (25). The differential response of brain natriuretic peptide reduction between men and women was not reported in these studies, but it is established from the MADIT-CRT trial that women experienced lower rates of HF or death at 4 years, a greater decrease in left ventricular end-diastolic volume, a greater increase in left ventricular ejection fraction at 1 year after CRT defibrillator implantation, and a greater reduction in ventricular tachycardia and ventricular fibrillation compared with men (26–28). These outcomes have yet to be studied in BAT, but it will be important to determine whether greater NT-proBNP reduction in women seen in this study ultimately portends improved morbidity and mortality, as may be the case with CRT.

### ADVERSE EVENTS AND PROCEDURAL COMPLICATIONS.

In this study, the safety of BAT as measured by MANCE-free and serious related adverse event rates was similar between men and women. Although the numbers are small, this contrasts with previous studies of device therapy (ICDs specifically) suggesting that women experience greater rates of post-procedural complications and adverse events. In a large registry-based study, women experienced more major (5.4% vs. 3.3%; p = 0.002) and minor (5.8% vs. 3.8%; p = 0.006) early complications with ICD insertion compared with men (29), but these data are based on large sample sizes in a real-world setting. In our study, 2 of the 4 subjects with MANCE (1 in a man, 1 in a woman) experienced infection in the neck requiring explantation, which is a complication related to the implantation or presence of the implanted system. Notably, in a retrospective analysis of 64,903 Medicare patients undergoing ICD generator implantation or revision, women who developed device infection after implantation had lower 5-year survival compared with men (67.3% vs. 72.9%; p < 0.02) (30). These differences in risk for adverse events in other procedural interventions for HFrEF highlight the importance of sex-based analyses, which must be further evaluated in post-market BAT trials as well.

### STUDY LIMITATIONS.

The major limitation of this study was the small number of women enrolled in BeAT-HF relative to men, as these comparisons were not prospectively powered. In many symptomatic domains (QoL score, exercise capacity, and functional status), women improved with BAT more than men, but the difference was not statistically significant, and it is possible that these differences in response might become significant in larger trials. In fact, the interaction p value between men and women became significant for QoL and NT-proBNP when the phase 2 data were included. Women receiving BAT plus GDMT, however, had significant decreases in NT-proBNP between treatment groups from baseline to 6 months, whereas in men, the decrease was not significant, except when phase 2 data were included. Although it is certainly possible that this difference may be spurious, NT-proBNP is a robust marker in this study, as it is both objective (in an unblinded trial) and a generally strong predictor of HF outcomes, as described. Larger studies are needed to confirm this finding.

Finally, the BeAT-HF trial thus far has not examined endpoints in morbidity and mortality or HF hospitalization; these are being studied in the now completed trial. Because baseline sex differences were established in a host of patient-centered outcomes, this prompts questions about whether women who are more symptomatic may be more likely to be identified and enrolled in clinical trials with a novel therapy such as BAT. Furthermore, these baseline differences appear across multiple facets of distress, from HF symptoms to anxiety and depressive reports. Fortunately, these baseline differences are not sustained across the course of the trial, and women experienced comparable benefits from the therapy.

## CONCLUSIONS

In the BeAT-HF trial, women had lower QoL measures at baseline compared with men but experienced significant improvement from baseline to 6 months in patient-centered variables such as QoL, exercise capacity, and functional status with BAT, similar to the response seen in men, with no significant difference in safety between sexes. Additionally, women had at least as much decrease in NT-proBNP compared with men. These preliminary findings are consistent with the response observed by sex to other GDMTs as well as CRT and suggest that women are likely to benefit from BAT at least as much as men.

## Supplementary Material

Supplement

## Figures and Tables

**CENTRAL ILLUSTRATION:**
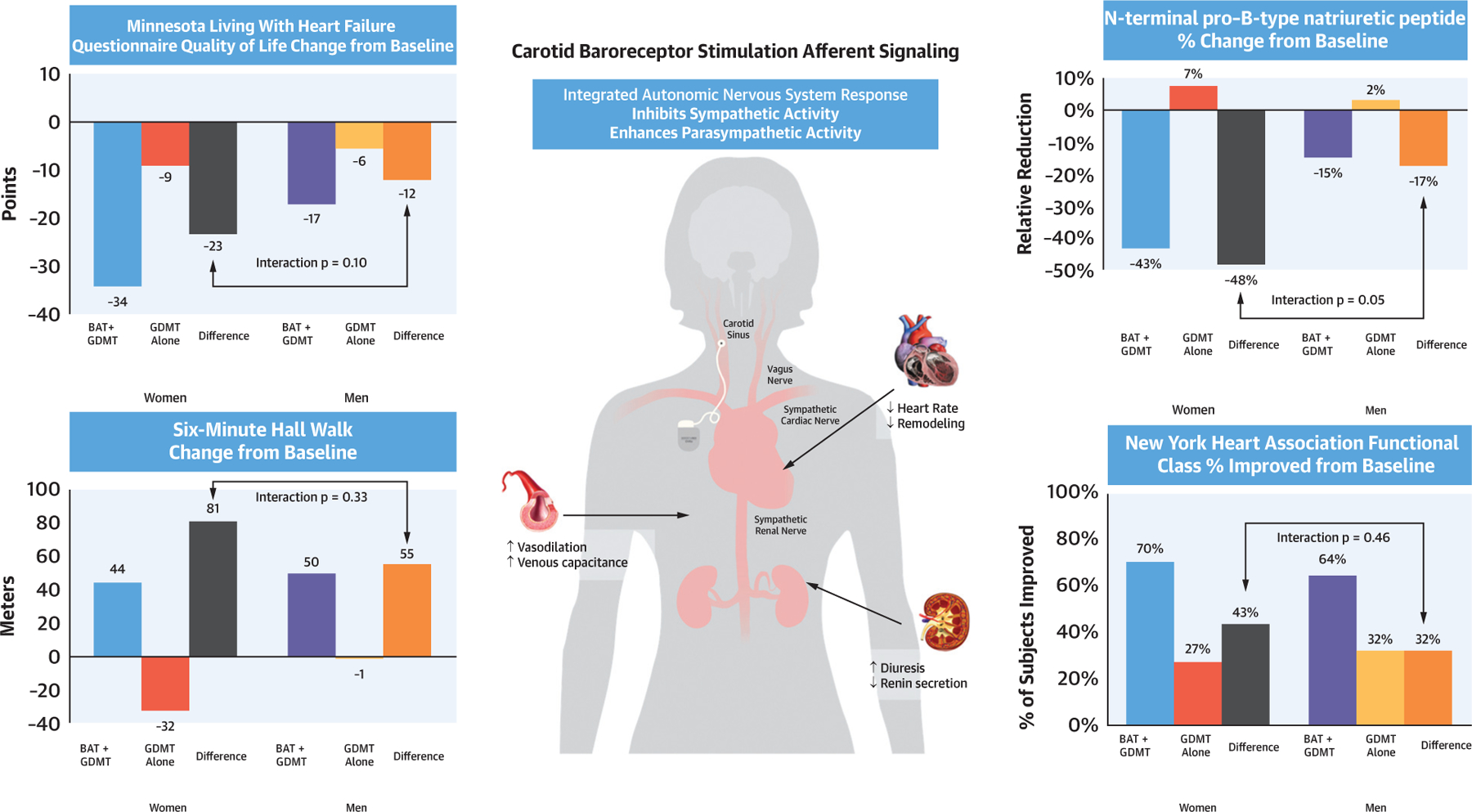
6-Month Results by Sex Women receiving baroreflex activation therapy (BAT) plus guideline-directed medical therapy (GDMT) had a significant benefit in the 3 primary endpoints and New York Heart Association functional class compared with GDMT alone, responding favorably to BAT, similar to men.

**TABLE 1:** Baseline Characteristics by Sex

	Female (n = 53)	Male (n = 211)	p Value
Baseline demographics			
Race			
Asian	1.9	1.9	1.00
Black or African American	21	16	0.41
White	70	74	0.60
Other/unknown	7.5	8.5	1.00
Age at screening (yrs)	61 ± 11	63 ± 11	0.31
Body mass index (kg/m^2^)	29 ± 5	31 ± 5	0.01
Systolic blood pressure (mm Hg)	122 ± 19	120 ± 16	0.39
Diastolic blood pressure (mm Hg)	73 ± 10	73 ± 10	0.63
Heart rate (beats/min)	77 ± 10	75 ± 11	0.19
Left ventricular ejection fraction (%)	28 ± 5	27 ± 6	0.08
Estimated glomerular filtration rate (ml/min/1.73 m^2^)	61 ± 17	63 ± 19	0.39
QRS interval (ms)	99 ± 14	112 ± 23	<0.001
Baseline comorbidities			
At least 1 heart failure hospitalization	40	48	0.36
Number of heart failure hospitalizations	0.5 ± 0.6	0.7 ± 1.0	0.08
Ischemic disease			
Coronary artery disease	53	68	0.05
Myocardial infarction	53	60	0.44
Coronary artery bypass grafting	11	27	0.02
Percutaneous coronary intervention	40	45	0.54
Cardiac arrhythmia			
Bradycardia	7.5	10.9	0.61
Tachycardia	34	34	1.00
Atrial fibrillation	32	37	0.63
Stroke or transient ischemic attack	21	20	1.00
Chronic kidney disease	21	25	0.59
Type II diabetes	49	47	0.88
Baseline heart failure treatments			
Number of medications	3.9 ± 1.3	4.1 ± 1.3	0.42
ACE inhibitors/ARBs			
Use	57	59	0.88
% use at recommended dose	28 ± 26	29 ± 25	0.91
Beta-blockers			
Use	94	95	0.74
% use at recommended dose	27 ± 26	29 ± 27	0.54
Diuretic agents			
Use	83	87	0.52
Ivabradine			
Use	3.8	3.3	1.00
Mineralocorticoid antagonist agents			
Use	38	47	0.28
% use at recommended dose	60 ± 57	57 ± 43	0.77
ARNIs			
Use	26	29	0.74
% use at recommended dose	32 ± 15	44 ± 26	0.09
ACE inhibitor/ARB or ARNI use	83	87	0.50
Implantable cardioverter defibrillator	77	79	0.85
Baseline endpoints			
N-terminal pro–B-type natriuretic peptide (pg/ml)	797 (516–967)	719 (473–1,058)	0.92
New York Heart Association functional class III	91	95	0.33
6-min walk distance (m)	289 ± 75	309 ± 70	0.08
Minnesota Living With Heart Failure Questionnaire score	62 ± 22	50 ± 24	0.01

Values are %, mean ± SD, or median (interquartile range).

ACE = angiotensin-converting enzyme; ARB = angiotensin receptor blocker; ARNI = angiotensin receptor–neprilysin inhibitor.

**TABLE 2:** Baseline Quality of Life and EuroQoL 5-Dimension Long by Sex

	Female (n = 53)	Male (n = 211)	p Value
Minnesota Living With Heart Failure Questionnaire quality of life			
Physical (8 questions)	28 ± 7.6	22 ± 10	<0.01
Emotional (5 questions)	15 ± 7.5	11 ± 7.6	<0.01
EuroQol 5-Dimension Long			
Overall health today	52 ± 20	58 ± 20	0.05
Mobility			0.69
I have no problems in walking	21	26	
I have slight problems in walking	32	31	
I have moderate problems in walking	36	36	
I have severe problems in walking	11.0	7.1	
Self-care			0.30
I have no problem washing or dressing myself	60	72	
I have slight problems washing or dressing myself	25	19	
I have moderate problems washing or dressing myself	13.0	8.1	
I have severe problems washing or dressing myself	1.9	0.9	
Usual activities			0.18
I have no problems doing my usual activities	7.5	19.0	
I have slight problems doing my usual activities	30	31	
I have moderate problems doing my usual activities	40	35	
I have severe problems doing my usual activities	15	12	
I am unable to do my usual activities	7.5	3.3	
Pain/discomfort			0.16
I have no pain or discomfort	19	31	
I have slight pain or discomfort	32	31	
I have moderate pain or discomfort	36	31	
I have severe pain or discomfort	11.0	4.3	
I have extreme pain or discomfort	1.9	2.4	
Anxiety/depression			<0.01
I am not anxious or depressed	25	45	
I am slightly anxious or depressed	32	27	
I am moderately anxious or depressed	32	21	
I am severely anxious or depressed	3.8	6.2	
I am extremely anxious or depressed	7.5	0.5	

Values are mean ± SD or %.

**TABLE 3:** 6-Month Quality of Life by Sex

	Female			Male			Interaction p Value
	BAT + GDMT	GDMT Alone	Difference	BAT + GDMT	GDMT Alone	Difference	
Minnesota Living With Heart Failure Questionnaire quality of life							
Physical (8 questions)	−15 ± 12	−4.2 ± 9.8	−10.0*	−7.2 ± 11	−3.0 ± 8.8	−4.1*	0.05
Emotional (5 questions)	−8.9 ± 7.5	−2.5 ± 6.5	−5.4*	−3.8 ± 6.8	−0.6 ± 6.1	−3.4*	0.28
EuroQoL 5-Dimension Long							
Overall health today	24 ± 20	9.2 ± 23	12.0*	14 ± 19	4.3 ± 18	9.5*	0.59
Mobility (% improved)	52	42	10	46	24	22*	0.36
Self-care (% improved)	39	12	27*	20	16	4	0.10
Usual activities (% improved)	70	23	47*	57	39	18*	0.06
Pain/discomfort (% improved)	48	27	21	42	23	19*	0.97
Anxiety/depression (% improved)	70	19	51*	38	17	21*	0.12

Values are mean ± SD or %.

* p ≤ 0.05.

BAT = baroreflex activation therapy; GDMT = guideline-directed medical therapy.

## PERSPECTIVES

### COMPETENCY IN MEDICAL KNOWLEDGE:

Women with HFrEF have greater symptom burden and more impaired QoL compared with men but experienced significant improvements in QoL, exercise capacity, functional status, and NT-proBNP with BAT, achieving a similar response to men.

### TRANSLATIONAL OUTLOOK:

Further studies are needed to assess the impact of BAT on morbidity and mortality in women and to determine whether response to therapy differs by sex.

## ABBREVIATIONS AND ACRONYMS

6MWD: 6-min walk distance
BAT: baroreflex activation therapy
CRT: cardiac resynchronization therapy
EQ-5D: EuroQol 5 Dimension Long
GDMT: guideline-directed medical therapy
HF: heart failure
HFrEF: heart failure with reduced ejection fraction
ICD: implantable cardioverter-defibrillator
MANCE: major adverse neurological and cardiovascular event(s)
MLWHQ: Minnesota Living With Heart Failure Questionnaire
NT-proBNP: N-terminal pro–B-type natriuretic peptide
NYHA: New York Heart Association
QoL: quality of life
