# Supplementary material for: Response by Sex in Patient-Centered Outcomes With Baroreflex Activation Therapy in Systolic Heart Failure
Source: JACC Heart Fail. Author manuscript; Available in PMC 2022 Feb 17. (PMC8852222; doi:10.1016/j.jchf.2021.01.012)
Supplement: Supplement [file NIHMS1774872-supplement-Supplement.docx]

Supplementary Appendix

Lindenfeld J, Gupta R, et al.

Table S1: 6-month clinically relevant measures of response by sex…………………..…............2
Table S2: System or procedure-related MANCE-free rates within 6 months of implant by sex…3

Table S3: System or procedure-related MANCE events within 6 months ………….…................4

Table S4: Serious related adverse events during study period by sex…………………………….5

Table S1: 6-month clinically relevant measures of response by sex

| **Endpoint Results** | **Female** | | | **Male** | | | **Female / Male**  **Interaction**  **p-value** |
| --- | --- | --- | --- | --- | --- | --- | --- |
|  | **BAT + GDMT (N=23)** | **GDMT Alone (N=26)** | **Difference** | **BAT + GDMT (N=97)** | **GDMT Alone (N=99)** | **Difference** |  |
| 6MHW | 44 ± 45 | -32 ± 118 | 81* | 50 ± 71 | -1.5 ± 78 | 55* | 0.33 |
| NYHA Improvement ≥ 1 Class | 70% | 27% | 43%* | 64% | 32% | 32%* | 0.46 |
| MLWHF QoL Score | -34 ± 27 | -9.0 ± 23 | -23* | -18 ± 24 | -5.5 ± 19 | -12* | 0.10 |
| **Clinically Relevant Responder** |  |  |  |  |  |  |  |
| 6MHW>10% | 70% | 20% | 50%* | 60% | 34% | 26%* | 0.13 |
| QoL>5 Points | 78% | 54% | 24% | 66% | 41% | 25%* | 0.87 |
| NYHA Improvement ≥1 Class | 70% | 27% | 43%* | 64% | 32% | 32%* | 0.46 |
| Clinically relevant response in ≥ 2 | 87% | 28% | 59%* | 68% | 29% | 39%* | 0.15 |
| Clinically relevant response in all 3 | 35% | 4% | 31%* | 28% | 9% | 19%* | 0.31 |
| **Super Responder** |  |  |  |  |  |  |  |
| 6MHW>20% | 39% | 20% | 19% | 33% | 18% | 15%* | 0.84 |
| QoL>10 Points | 78% | 42% | 36%* | 57% | 34% | 23%* | 0.34 |
| NYHA Improvement to Class I | 22% | 4% | 18% | 14% | 2% | 12%* | 0.91 |
| Super response in ≥ 2 | 43% | 8% | 35%* | 24% | 11% | 13%* | 0.21 |

* p-value<0.05

Table S2: System or procedure-related MANCE-free rates within 6 months of implant by sex

|  | **Total Number of Subjects** | **Number of Subjects MANCE-Free Rate** | **MANCE-Free Rate** | **Interaction P-value** |
| --- | --- | --- | --- | --- |
| Male | 101 | 98 | 97% | 0.57 |
| Female | 24 | 23 | 96% |  |

Table S3: System or procedure-related MANCE events within 6 months

| **MANCE Event** | **Gender** | **Event** | **Days since Implant** | **Intervention** | **Outcome** | **Procedure Related** | **System Related** |
| --- | --- | --- | --- | --- | --- | --- | --- |
| Acute Decompensated HF | Male | Acute decompensated heart failure | 1 | HF medications change or administration: IV administered; Hospital visit or admission | Recovered, no residual effects | Related | Not related |
| Infection Requiring Explant | Female | Postoperative Wound Infection | 6 | Other medication change or administration; Change to Neo System: Surgical Intervention; Hospital visit or admission | Recovered, no residual effects | Related | Related |
|  | Male | Device Infection | 25 | Other medication change or administration; Diagnostic testing: Wound culture of right neck drainage; Change to Neo System: Surgical Intervention; Hospital visit or admission | Recovered, no residual effects | Related | Related |
| Stroke | Male | Acute left-sided CVA | 11 | Diagnostic testing: Head CT; Hospital visit or admission | Recovered, with residual effects | Related | Not related |

Table S4: Serious related adverse events in the first six months post implant by sex

| **Relation** | **Female** | **Male** |
| --- | --- | --- |
|  | **N=24 Implants** | **N=101 Implants** |
| System | 0 (0%) | 0 (0%) |
| Procedure | 1 (4%) | 5 (5%) |
| System and Procedure | 1 (4%) | 1 (1%) |
| **Total** | **1 (4%)** | **6 (6%)** |
